# Genome-wide analysis of diamondback moth, *Plutella xylostella* L., from *Brassica* crops and wild host plants reveals no genetic structure in Australia

**DOI:** 10.1038/s41598-020-68140-w

**Published:** 2020-07-21

**Authors:** Kym D. Perry, Michael A. Keller, Simon W. Baxter

**Affiliations:** 1 School of Agriculture Food and Wine, University of Adelaide, Adelaide, 5005 Australia; 2 Entomology Unit, South Australian Research and Development Institute, Adelaide, 5001 Australia; 3 School of BioSciences, University of Melbourne, Melbourne, 3010 Australia

**Keywords:** Ecology, Genetics

## Abstract

Molecular studies of population structure can reveal insight into the movement patterns of mobile insect pests in agricultural landscapes. The diamondback moth, *Plutella xylostella* L., a destructive pest of *Brassica* vegetable and oilseed crops worldwide, seasonally colonizes winter canola crops in southern Australia from alternative host plant sources. To investigate movement, we collected 59 *P. xylostella* populations from canola crops, *Brassica* vegetable and forage crops and brassicaceous wild host plants throughout southern Australia in 2014 and 2015 and genotyped 833 individuals using RAD-seq for genome-wide analysis. Despite a geographic sampling scale > 3,000 km and a statistically powerful set of 1,032 SNP markers, there was no genetic differentiation among *P. xylostella* populations irrespective of geographic location, host plant or sampling year, and no evidence for isolation-by-distance. Hierarchical STRUCTURE analysis at *K* = 2–5 showed nearly uniform ancestry in both years. Cluster analysis showed divergence of a small number of individuals at several locations, possibly reflecting an artefact of sampling related individuals. It is likely that genetic homogeneity within Australian *P. xylostella* largely reflects the recent colonization history of this species but is maintained through some level of present gene flow. Use of genome-wide neutral markers was uninformative for revealing the seasonal movements of *P. xylostella* within Australia, but may provide more insight in other global regions where the species has higher genetic diversity.

## Introduction

Mobile insect pests regularly colonize annual crops from alternative host plant sources^1–3^. Protecting crops from attack by these pests is difficult for pest managers due to the unpredictable nature of seasonal outbreaks, particularly when insecticide resistant genotypes are present^4,5^. For mobile insect pests, dispersal among crop and non-crop host plant resources influences both the seasonal dynamics and genetic background of pest populations, with direct consequences for pest management^6–9^. Molecular studies of population structure and gene flow can potentially provide insight into patterns of insect movement in agricultural landscapes^10,11^.

The diamondback moth, *Plutella xylostella* L., is the most destructive pest of brassicaceous crops worldwide^12,13^. It attacks *Brassica* vegetable crops throughout tropical and temperate regions^14^ and in recent decades has become a significant pest of canola crops in temperate regions^4,15–18^. The propensity of *P. xylostella* to evolve insecticide resistance rapidly and a lack of alternative control options has led it to evolve resistance to most pesticides^4^. Within Australia, *P. xylostella* has been a major pest of *Brassica* vegetable crops since the late 1800s^19^ and a sporadic but damaging pest of canola crops since the 1990s following a dramatic expansion of canola production^18^. Approximately 3 million hectares of canola is grown annually under a Mediterranean climate in southern Australia^20^, providing vast host resources for *P. xylostella* from crop planting in autumn to maturity in late spring. Intermittent outbreaks of *P. xylostella* in canola during spring cause substantial crop losses^18,21^. Commercial *Brassica* vegetable crops are grown continuously in horticultural areas surrounding the major urban centres in each Australian state, occupying a total area less than 1% of total canola plantings^22^. Other host plants for *P. xylostella* include *Brassica* forage crops grown during spring and summer as stock feed, and a diversity of introduced and native wild brassicaceous species distributed over vast areas and proliferated by rainfall. In *Brassica* vegetable crops, limited *P. xylotella* dispersal and intense insecticide use targeting this insect can lead to elevated levels of insecticide resistance in local *P. xylostella* populations^6,22,23^. In canola-growing areas, the highly seasonal availability of host plants compels *P. xylostella* to move regularly among crops and other brassicaceous host plants, which tends to homogenise levels of insecticide resistance^24^. Estimating gene flow among local populations of *P. xylostella* between host plant types and identifying source populations of *P. xylostella* that seasonally infest Australian canola are essential to facilitate forecasting of seasonal pest pressure and inform insecticide resistance management^13,18,19^.

Various molecular markers have been used to investigate population structure in *P. xylostella*, including allozymes, ISSRs, microsatellites and mtDNA. *Plutella xylostella* populations from different continents are clearly differentiated^19,25,26^, but a lack of genetic structure among populations within parts of Asia^10,27,28^, the USA^29,30^ and Australasia^19,31^ implies regular intermixing at an intra-continental level. Many population genetic studies of *P. xylostella* have been difficult to interpret due to limited sampling^32^ and few studies have sampled at a sufficient spatiotemporal scale or resolution to investigate movement at a landscape scale. However, two studies successfully identified the seasonal migration pathways of *P. xylostella* in China through extensive field sampling and analysis of both microsatellite markers and geographic variation in mtDNA haplotype frequencies^10,28^. Inferences from genetic data were corroborated by light trapping^33^.

Within Australia, Endersby et al.^19^ found no differentiation at six microsatellite loci among 17 populations across Australia and one from New Zealand, despite a sampling scale spanning > 5,000 km. These Australasian populations were clearly differentiated from populations collected in Asia and Africa^19^. Australian *P. xylostella* has low genetic diversity consistent with a founder effect^19,26,31,34,35^. Present levels of gene flow among Australian *P. xylostella* populations remain to be resolved because genetic homogeneity could reflect co-ancestry^18^, and because the statistical power of six microsatellites to detect weak population structure was uncertain. Furthermore, inconsistent patterns of population structure reported among *P. xylostella* collected from eastern Australia^25,36^ may reflect the presence of a cryptic species, *Plutella australiana*, among analysed samples^35,37^.

The revolution in massively parallel sequencing technologies^38^ and associated genotyping methods has facilitated genome-wide genetic marker sets and brought unprecedented resolution to questions of population structure^39,40^. Restriction-site-associated DNA sequencing (RAD-seq)^41^ enables sequencing of targeted short regions across the genome, allowing simultaneous discovery and genotyping of single nucleotide polymorphisms (SNPs) in model and non-model species^42,43^. The ability to sequence orthologous regions across multiple individuals at high sequencing coverage makes it possible to confidently genotype SNPs and generate high density markers for population genetic studies^40,44^. Microsatellites remain popular for population genetic studies due to high polymorphism^45^, but can be outperformed by large SNP panels in resolving population structure^46,47^, with several examples in insects^48,49^. RAD-seq has genotyped thousands of SNPs in *P. xylostella*^50^ and resolved species-level nuclear divergence between cryptic Australian *Plutella* species^35^, suggesting potential for this method to provide insight into the movement patterns of *P. xylostella*.

Here, we examined whether geographic, host plant-related or temporal population genetic structure exists among geographically distinct populations of *P. xylostella* in Australia. Samples were collected from canola crops, *Brassica* vegetable crops, *Brassica* forage crops and wild brassicaceous plant species throughout southern Australia and in two consecutive years to facilitate temporal comparisons. After molecular species identification, *P. xylostella* individuals were genotyped across genome-wide sites using RAD sequencing for population genetics analysis.

## Results

### Sample collection

**Figure 1:**
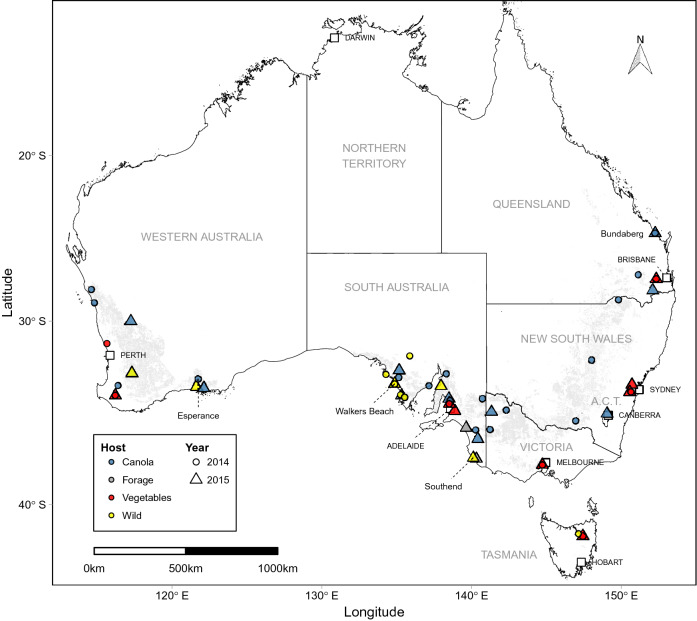
Geographic locations of 59 *P. xylostella* populations collected in Australia in 2014 and 2015 and sequenced using RAD-seq. Collections from different *Brassica* host types are represented by different colours. Canola is grown in dryland cropping areas of southern Australia, represented in grey shading.

*Plutella* species were collected from different *Brassica* host plants and locations throughout southern Australia in 2014 and 2015 (Fig. 1). After species identification using PCR-RFLP, 909 *P. xylostella* individuals from 60 locations, 32 in 2014 and 28 in 2015, were retained for analysis (Table 1). In total, 29 populations were collected from canola crops, 15 from *Brassica* vegetable crops, three from *Brassica* forage crops and 13 from brassicaceous weeds. Of these, 52 populations were collected in spring and seven in autumn. Seven locations were sampled in both 2014 and 2015 to facilitate a temporal analysis, of which five locations were *Brassica* vegetable crops from the major *Brassica* vegetable production areas in each Australian state (Fig. 1). Sex was determined for the 681 pupal individuals (82% of all individuals) but not larvae. The overall sex ratio was not different from 1:1 (364 males, 317 females, $\chi ^2=3.2438$, *p* = 0.0717) and most populations had a reasonably balanced sex ratio (Table 1).

**Table 1:** Summary of *P. xylostella* collections from Australia.

Location^1^	Collection date	Coordinates	Host plant	No. sequenced^2^		
				Total		
Boomi NSW	Sep-2014	28.76° S 149.81° E	Canola	10	7	3
Ginninderra NSW	Oct-2015	35.19° S 149.05° E	Canola	14	6	8
Henty NSW	Oct-2014	35.60° S 146.95° E	Canola	16	9	7
Narromine NSW	Sep-2014	32.22° S 148.03° E	Canola	16	7	3
Richmond NSW	Oct-2015	33.60° S 150.71° E	Cabbage	16	7	9
Werombi NSW	Nov-2014	33.99° S 150.64° E	*Brassica* vegetables	10	5	4
Werombi NSW	Oct-2015	34.00° S 150.56° E	Kale	9	4	5
Bundaberg QLD	Oct-2014	24.80° S 152.26° E	Canola	12	7	3
Bundaberg QLD	Sep-2015	24.80° S 152.26° E	Canola	16	5	10
Dalby QLD	Sep-2014	27.28° S 151.13° E	Canola	14	6	8
Gatton QLD	Oct-2014	27.54° S 152.33° E	Broccoli	14	6	5
Gatton QLD	Nov-2015	27.54° S 152.33° E	Broccoli	14	7	7
Warwick QLD	Oct-2015	28.21° S 152.11° E	Canola	14	8	6
Calca SA	Apr-2014	33.02° S 134.28° E	Sand rocket, wall rocket	9	4	1
Cocata SA	Sep-2014	33.20° S 135.13° E	Canola	16	4	7
Colebatch SA	Feb-2015	35.97° S 139.66° E	*Brassica* forage	12	4	5
Cowell SA	Sep-2014	33.66° S 137.16° E	Canola	16	6	0
Keith SA	Oct-2014	36.09° S 140.29° E	Canola	12	5	6
Littlehampton SA	Sep-2015	35.06° S 138.90° E	Brussels sprouts	9	3	3
Loxton SA	Sep-2014	34.37° S 140.72° E	Canola	16	8	8
Mallala SA	Sep-2015	34.38° S 138.50° E	Canola	15	9	6
Millicent SA	Apr-2015	37.61° S 140.34° E	Canola	9	2	0
Minnipa SA	Oct-2015	32.81° S 135.16° E	Canola	16	8	6
Moonaree SA	Aug-2014	31.99° S 135.87° E	Ward’s weed	16	0	0
Mt Hope SA	Sep-2014	34.14° S 135.33° E	Canola	16	7	6
Mt Hope SA	Sep-2015	34.20° S 135.34° E	Canola	16	7	9
Padthaway SA	Oct-2015	36.56° S 140.43° E	Canola	14	9	5
Picnic Beach SA	Apr-2014	34.17° S 135.27° E	Sea rocket	8	0	2
Redbanks SA	Oct-2014	34.49° S 138.59° E	Canola	15	3	6
Southend SA	Apr-2015	37.57° S 140.12° E	Sea rocket	16	8	8
Tintinara SA	Oct-2015	35.97° S 139.66° E	*Brassica* forage	16	8	8
Virginia SA	Oct-2014	34.64° S 138.54° E	Broccoli	16	4	1
Virginia SA	Sep-2015	34.64° S 138.54° E	Cabbage	16	10	5
Walkers Beach SA	Sep-2014	33.55° S 134.86° E	Sea rocket	16	7	6
Walkers Beach SA	Mar-2015	33.55° S 134.86° E	Sea rocket	16	8	8
Walkers Beach SA	Sep-2015	33.55° S 134.86° E	Sea rocket	12	6	6
Wirrabara SA	Oct-2014	32.99° S 138.31° E	Canola	15	5	3
Wokurna SA	Sep-2015	33.67° S 137.96° E	Wild radish	16	9	4
Wurramunda SA	Apr-2014	34.30° S 135.56° E	Volunteer canola	16	9	7
Deddington TAS	Nov-2014	41.59° S 147.44° E	Kale	12	6	6
Deddington TAS	Nov-2015	41.59° S 147.44° E	Cauliflower	16	5	7
Launceston TAS	Nov-2014	41.47° S 147.14° E	Wild mustard	16	9	7
Cowangie VIC	Oct-2015	35.10° S 141.33° E	Canola	15	7	5
Ouyen VIC	Sep-2014	35.00° S 142.31° E	Canola	15	9	5
Werribee VIC	Oct-2014	37.94° S 144.73° E	Cauliflower	16	2	3
Werribee VIC	Nov-2015	37.94° S 144.73° E	Cauliflower	13	7	6
Yanac VIC	Sep-2014	36.06° S 141.25° E	Canola	12	6	6
Boyup Brook WA	Sep-2014	33.64° S 116.40° E	Canola	15	5	3
Dalyup WA	Oct-2015	33.72° S 121.64° E	Wild radish	16	9	7
Esperance WA	Sep-2014	33.29° S 121.76° E	Canola	12	2	1
Esperance WA	Oct-2015	33.79° S 122.13° E	Canola	15	7	8
Gingin WA	Dec-2014	31.28° S 115.65° E	Red cabbage	16	10	6
Kalannie WA	Sep-2015	30.00° S 117.25° E	Canola	16	8	8
Manjimup WA	Dec-2014	34.18° S 116.23° E	Chinese cabbage	9	5	4
Manjimup WA	Nov-2015	34.18° S 116.23° E	*Brassica* vegetables	13	3	9
Narrogin WA	Oct-2015	32.96° S 117.33° E	Canola	13	7	6
Narrogin WA	Oct-2015	32.95° S 117.32° E	Wild radish, volunteer canola	16	8	8
Northampton WA	Sep-2014	28.16° S 114.63° E	Canola	16	9	4
Walkaway WA	Sep-2014	28.94° S 114.83° E	Canola	16	3	4

^1^Australian states: *NSW* New South Wales, *QLD* Queensland, *SA* South Australia, *TAS* Tasmania, *VIC* Victoria, *WA* Western Australia.

^2^Total includes males and females (pupae) and unknown sex (larvae)

### Read filtering and variant calling

RAD-seq was performed for 909 *P. xylostella* individuals from 60 collection locations, including 15 individuals randomly selected from different library pools and sequenced as technical duplicates to check the robustness of genotype calls. Illumina sequencing yielded 2.36 billion raw sequence reads after de-multiplexing. Following read trimming and filtering, mapping, genotype calling and hard-filtering, we excluded 50 individuals with greater than 60% missing data, which was largely due to low sequencing depth (Supplementary Fig. S1), then excluded the 15 technical duplicates and a population with only two individuals remaining. Nine individuals with unusually high levels of polypmorphism and investigated using mtDNA amplicon sequencing were found to be contaminated and were excluded. Genotyping and hard-filtering steps were then repeated for the remaining 833 individuals across 59 population samples, including 434 individuals from 31 populations collected in 2014 and 399 individuals from 28 populations collected in 2015. Hard-filtering retained 590,086 confidently-called (GQ $\geqslant$ 30) variant and invariant sites at a mean depth of 33.4 per individual, and a subset of 1,032 widely-dispersed (to avoid linkage bias) bi-allelic SNP variants at a mean depth of 34.0 per individual, for downstream analyses. In reference-aligned SNP datasets with read depth > 30, genotyping error rates are expected to be < 0.01^51^. The datasets for 2014 and 2015 were analysed separately.

For the 15 technical duplicates, the VCF output from HaplotypeCaller was hard-filtered using our parameters to retain the 30 samples and a set of 1,473 widely-dispersed bi-allelic SNP variants. Principal components analysis showed that sample pairs group closely together, indicating that genotype calls were highly consistent (Supplementary Fig. S2).

### Genetic diversity statistics

Population genetic diversity was estimated using the 590,086 variant and invariant sites. The mean observed heterozygosity per population averaged 0.0092 ± 0.0002 SD (range = 0.0088, 0.0097) across the 59 populations and showed little variation across populations collected from different years (2014, *n* = 31 and 2015, *n* = 28), host plant types (canola, *n* = 30, *Brassica* vegetable crops, *n* = 15, *Brassica* forage crops, *n* = 2, and wild brassicas, *n* = 12) or seasons (autumn, *n* = 7 or spring, *n* = 52). In general, observed heterozygosity was lower than expected as shown by mostly positive $F_{\text{IS}}$ values, suggesting some inbreeding (Tables 2, 3, Supplementary Fig. S3). The population from Southend 2015 had reduced gene diversity and fewer private sites relative to other populations. Across the 1,032 SNPs, observed heterozygosity and gene diversity within each year showed reasonable agreement (Tables 2, 3, Supplementary Fig. S3), indicating allele frequencies at these loci are in Hardy–Weinberg proportions. Again, for this marker set, the Southend 2015 population had the lowest genetic diversity among populations, contributing to a negative $F_{\text{IS}}$ value.

**Table 2:** Population statistics for all 590,086 confidently-called variant and invariant sites, and a subset of 1,032 hard-filtered SNP loci, for 31 *P. xylostella* populations collected from Australia in 2014.

Population	All variant and invariant sites									1,032 SNP variants				
	*N*	Sites	Site depth	SNPs	Indels	Private sites	$H_{\text {O}}$	$H_{\text {S}}$	$F_{\text{IS}}$	*N*	Site depth	$H_{\text {O}}$	$H_{\text {S}}$	$F_{\text{IS}}$
Boomi NSW	9.5	562,586	38	8,590	1,653	16	0.0090	0.0095	0.0398	9.3	38	0.2096	0.2057	− 0.0204
Henty NSW	15.3	564,870	33	8,418	1,618	14	0.0092	0.0096	0.0496	14.5	33	0.2042	0.2052	0.0048
Narromine NSW	15.0	553,119	30	8,216	1,558	18	0.0093	0.0097	0.0382	13.8	31	0.2077	0.2055	− 0.0081
Werombi NSW	9.3	550,438	26	8,086	1,518	16	0.0095	0.0097	0.0179	8.4	28	0.2120	0.2074	− 0.0244
Bundaberg QLD	11.3	557,174	38	8,338	1,578	16	0.0091	0.0096	0.0451	10.7	38	0.2050	0.2030	− 0.0105
Dalby QLD	13.5	567,483	36	8,495	1,630	16	0.0093	0.0096	0.0402	12.9	36	0.2095	0.2086	− 0.0020
Gatton QLD	12.9	543,911	28	7,938	1,491	12	0.0095	0.0096	0.0152	12.0	29	0.2160	0.2030	− 0.0459
Calca SA	8.3	546,958	40	8,250	1,588	30	0.0095	0.0099	0.0354	7.5	40	0.2208	0.2205	− 0.0076
Cocata SA	15.0	553,050	37	8,119	1,560	13	0.0093	0.0097	0.0367	13.9	37	0.2014	0.2031	0.0040
Cowell SA	15.1	557,172	32	8,276	1,578	17	0.0094	0.0098	0.0378	13.8	32	0.2112	0.2094	− 0.0077
Keith SA	10.8	532,878	24	7,599	1,434	18	0.0097	0.0098	0.0104	9.3	26	0.2172	0.2065	− 0.0385
Loxton SA	15.3	564,013	42	8,590	1,639	22	0.0091	0.0096	0.0540	15.0	42	0.1965	0.2022	0.0182
Moonaree SA	15.2	560,304	33	8,354	1,595	17	0.0094	0.0097	0.0385	14.0	34	0.2142	0.2082	− 0.0207
Mt Hope SA	15.2	560,623	37	8,262	1,593	14	0.0092	0.0096	0.0459	14.3	37	0.1986	0.2014	0.0067
Picnic Beach SA	7.5	550,986	44	8,125	1,561	33	0.0097	0.0099	0.0128	6.4	44	0.2233	0.2144	− 0.0400
Redbanks SA	13.2	519,055	36	7,591	1,417	17	0.0091	0.0096	0.0536	12.9	36	0.2084	0.2056	− 0.0106
Virginia SA	15.3	564,927	32	8,437	1,620	16	0.0092	0.0097	0.0467	14.5	33	0.2087	0.2063	− 0.0072
Walkers Beach SA	15.2	560,602	35	8,371	1,599	21	0.0091	0.0097	0.0518	14.8	35	0.2002	0.2018	0.0013
Wirrabara SA	13.6	536,022	38	7,888	1,512	13	0.0091	0.0096	0.0541	13.1	38	0.2031	0.2021	− 0.0032
Wurramunda SA	15.3	565,796	41	8,630	1,651	20	0.0091	0.0095	0.0427	15.1	41	0.2001	0.2030	0.0117
Deddington TAS	11.0	539,076	25	7,792	1,454	17	0.0097	0.0098	0.0171	9.6	26	0.2182	0.2109	− 0.0292
Launceston TAS	15.1	557,084	33	8,318	1,602	15	0.0093	0.0097	0.0406	14.1	34	0.2110	0.2072	− 0.0120
Ouyen VIC	14.2	557,715	34	8,246	1,589	18	0.0094	0.0097	0.0350	13.2	34	0.2082	0.2061	− 0.0072
Werribee VIC	15.2	560,377	35	8,411	1,599	17	0.0092	0.0097	0.0507	14.4	35	0.2093	0.2096	0.0024
Yanac VIC	11.6	569,684	39	8,534	1,638	17	0.0090	0.0096	0.0520	11.2	39	0.1984	0.2044	0.0168
Boyup Brook WA	14.4	566,791	35	8,510	1,630	14	0.0092	0.0096	0.0365	13.8	35	0.2089	0.2031	− 0.0219
Esperance WA	11.2	550,595	33	8,156	1,551	30	0.0095	0.0097	0.0182	10.1	34	0.2128	0.2069	− 0.0289
Gingin WA	15.2	559,983	35	8,353	1,590	14	0.0089	0.0096	0.0710	14.8	35	0.1959	0.2014	0.0160
Manjimup WA	8.4	553,540	27	8,188	1,551	14	0.0095	0.0096	0.0107	7.7	28	0.2061	0.2043	− 0.0173
Northampton WA	15.4	568,041	38	8,558	1,646	16	0.0090	0.0095	0.0543	15.0	38	0.1949	0.2007	0.0158
Walkaway WA	15.2	560,808	38	8,311	1,591	16	0.0090	0.0096	0.0619	14.9	39	0.1978	0.2026	0.0207

*N*, number of individuals genotyped per locus; $H_{\text {O}}$, observed heterozygosity; $H_{\text {S}}$, gene diversity; $F_{\text{IS}}$, Nei’s inbreeding coefficient.

**Table 3:** Population statistics for all 590,086 confidently-called variant and invariant sites, and a subset of 1,032 hard-filtered SNP loci, for 28 *P. xylostella* populations collected from Australia in 2015.

Population	All variant and invariant sites									1,032 SNP variants				
	*N*	Sites	Site depth	SNPs	Indels	Private sites	$H_{\text {O}}$	$H_{\text {S}}$	$F_{\text{IS}}$	*N*	Site depth	$H_{\text {O}}$	$H_{\text {S}}$	$F_{\text{IS}}$
Goulburn NSW	13.3	559,574	33	8,318	1,574	15	0.0090	0.0096	0.0545	12.9	34	0.1948	0.1985	0.0153
Richmond NSW	15.2	560,841	37	8,349	1,595	14	0.0089	0.0096	0.0634	14.9	38	0.1983	0.1994	0.0005
Werombi NSW	8.5	556,788	42	8,473	1,605	13	0.0088	0.0094	0.0438	8.3	42	0.2033	0.2008	− 0.0127
Bundaberg QLD	14.5	536,071	25	7,777	1,472	16	0.0095	0.0097	0.0371	13.3	26	0.2131	0.2065	− 0.0247
Gatton QLD	12.7	533,431	27	7,674	1,438	18	0.0095	0.0098	0.0365	11.4	28	0.2129	0.2116	− 0.0019
Warwick QLD	13.2	555,382	33	8,328	1,590	20	0.0092	0.0097	0.0461	12.3	33	0.2012	0.2020	− 0.0015
Colebatch SA	11.5	565,070	31	8,449	1,612	17	0.0091	0.0096	0.0445	11.0	32	0.2023	0.2036	0.0019
Littlehampton SA	8.4	551,419	38	8,463	1,616	19	0.0091	0.0097	0.0495	8.2	38	0.2039	0.2068	0.0005
Mallala SA	13.9	545,678	28	8,012	1,526	20	0.0093	0.0096	0.0350	13.1	29	0.2090	0.2060	− 0.0133
Millicent SA	8.1	532,566	32	8,054	1,539	15	0.0089	0.0096	0.0607	7.8	32	0.2020	0.2033	0.0010
Minnipa SA	15.3	563,734	34	8,433	1,608	17	0.0091	0.0095	0.0506	14.6	34	0.2063	0.2061	− 0.0004
Mt Hope SA	14.9	551,211	30	8,117	1,542	18	0.0093	0.0097	0.0417	13.4	31	0.2164	0.2101	− 0.0211
Padthaway SA	12.6	529,583	30	7,804	1,488	16	0.0091	0.0096	0.0513	12.0	30	0.2042	0.2061	0.0005
Southend SA	15.3	563,465	34	8,365	1,597	6	0.0093	0.0091	− 0.0131	14.4	34	0.2099	0.1953	− 0.0598
Tintinara SA	14.6	539,118	25	7,853	1,499	14	0.0096	0.0097	0.0278	13.4	26	0.2093	0.2026	− 0.0202
Virginia SA	15.4	567,767	35	8,548	1,648	15	0.0092	0.0096	0.0485	14.9	35	0.2053	0.2058	− 0.0023
Walkers Beach SA	15.3	564,509	36	8,455	1,627	15	0.0091	0.0094	0.0407	14.8	36	0.1968	0.1958	− 0.0067
Walkers Beach SA	11.3	557,146	26	8,246	1,564	15	0.0095	0.0098	0.0276	10.4	27	0.2099	0.2064	− 0.0137
Wokurna SA	15.1	558,036	33	8,246	1,579	19	0.0094	0.0097	0.0379	14.0	34	0.2120	0.2105	− 0.0098
Deddington TAS	15.3	565,674	40	8,492	1,630	16	0.0091	0.0096	0.0544	15.0	40	0.2022	0.2044	0.0054
Cowangie VIC	14.4	565,260	35	8,457	1,612	19	0.0092	0.0096	0.0445	13.7	35	0.2100	0.2083	− 0.0051
Werribee VIC	11.9	538,661	25	7,878	1,469	16	0.0093	0.0097	0.0416	11.0	27	0.2091	0.2050	− 0.0176
Dalyup WA	15.3	562,709	32	8,452	1,624	17	0.0092	0.0097	0.0459	14.4	32	0.2027	0.2048	0.0017
Esperance WA	13.5	532,826	27	7,718	1,460	20	0.0096	0.0098	0.0239	12.2	28	0.2109	0.2043	− 0.0262
Kalannie WA	15.3	564,388	33	8,410	1,614	17	0.0091	0.0096	0.0496	14.6	34	0.2046	0.2065	0.0009
Manjimup WA	12.3	556,387	36	8,274	1,567	18	0.0091	0.0096	0.0517	12.0	37	0.2007	0.2032	0.0095
Narrogin WA	11.9	541,517	29	7,947	1,512	16	0.0094	0.0097	0.0376	11.1	30	0.2115	0.2104	− 0.0048
Narrogin WA	15.1	557,879	34	8,284	1,582	18	0.0090	0.0096	0.0579	14.7	35	0.1969	0.2011	0.0125

*N*, number of individuals genotyped per locus; $H_{\text {O}}$, observed heterozygosity; $H_{\text {S}}$, gene diversity; $F_{\text{IS}}$, Nei’s inbreeding coefficient.

### Power analysis

The power analysis indicated that our SNP marker loci had a high level of statistical power to detect even weak population structure. The 1,032 SNP loci had 100% probability of detecting true $F_{\text {ST}}$ values of 0.0027 or 0.0056 (Supplementary Table S1), corresponding to the estimated global $F_{\text {ST}}$ values for the 2014 and 2015 datasets.

### Population differentiation

**Figure 2:**
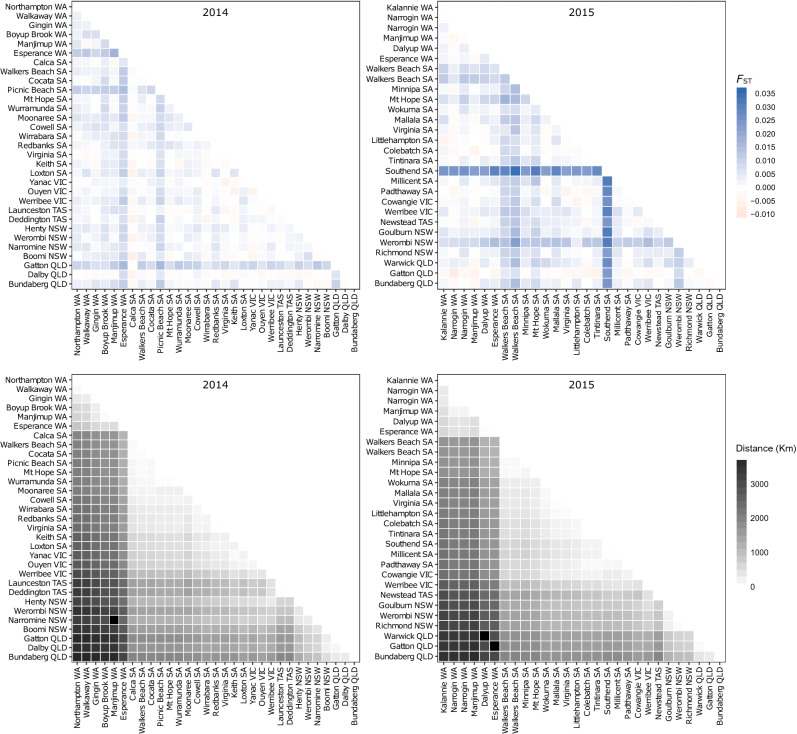
Heat maps showing pairwise comparisons of genetic distance measured as Weir and Cockerham’s (1984) $F_{\text {ST}}$ (top panels) and geographic distance in kilometres (bottom panels) among *P. xylostella* populations collected from Australia in 2014 (left panels) and 2015 (right panels). Within each year, populations on *x* and *y*-axes are sorted geographically from north-western to north-eastern Australia in an arc following the southern coast. Visual comparison of the $F_{\text {ST}}$ and geographic distance heat maps within each year shows no congruence between genetic and geographic distance among population pairs in 2014 or 2015.

The global estimates of $F_{\text {ST}}$ calculated using 1,032 SNPs were not significantly different from zero in either 2014 ($F_{\text {ST}}=0.0027$, 99% CL $=\,-$ 0.0043, 0.0107) or 2015 ($F_{\text {ST}}=0.0056$, 99% CL $=\,-$ 0.0019, 0.0138), indicating a lack of genetic differentiation among populations within years. Pairwise $F_{\text {ST}}$ values were generally very low, ranging from 0.0065 to 0.0178 (mean 0.0029 ± 0.0040 SD) in 2014 and − 0.0077 to 0.0344 (mean 0.0054 ± 0.0075 SD) in 2015 (Fig. 2). After correction for multiple comparisons (2014: *n* = 465 comparisons, 2015: *n* = 365 comparisons), no pairwise $F_{\text {ST}}$ values were significant at the target $\alpha =0.05$ level, indicating a lack of genetic differentiation among *P. xylostella* populations collected within a single year. The highest pairwise $F_{\text {ST}}$ values were associated with the Southend 2015 population, ranging from 0.0221 to 0.0344 (mean 0.0265 ± 0.0035 SD, *n* = 27 comparisons), indicating allele frequencies in this population were the most divergent from other populations (Fig. 2). AMOVA analysis using 1,032 SNPs indicated a lack of any spatial, temporal or host-plant related genetic structure among populations (Table 4). In model A, where populations were divided into years and *Brassica* host types, > 99% of variance was found within populations with negligible variance among populations explained by year or host type. Similarly, in model B, where seven locations were sampled in both years, > 99% of variance was found within populations. These results precluded interpretation of whether there was more spatial or temporal variance among populations.

Under isolation by distance, geographic and genetic distances should be positively correlated^52^. Populations were collected across geographic distances of up to 3,756 km (Northampton WA/Bundaberg QLD) in 2014 (mean distance 1,323 ± 960 SD km) and 3,624 kilometres (Manjimup WA/Bundaberg QLD) in 2015 (mean distance 1,263 ± 917 SD km). Given the vast sampling scale, we expected higher $F_{\text {ST}}$ values at greater geographic distances between population pairs, however heat maps revealed no such pattern (Fig. 2). Mantel tests confirmed a lack of genetic isolation by distance in both 2014 (Mantel’s *r* = 0.1136, *p* = 0.1316) and 2015 (Mantel’s *r* = − 0.0901, *p* = 0.8222) datasets, indicating that *P. xylostella* populations in close proximity or separated by thousands of kilometres were equally differentiated.

**Table 4:** Analysis of molecular variance under two hierarchical model structures.

AMOVA summary					
Source	df	SS	MS	Est. var.	%
**Model A**					
Year	1	141.844	141.844	0.015	0.013
Host	5	651.478	130.296	0.049	0.043
Population	52	6,487.275	124.755	0.902	0.798
Error	774	86,712.706	112.032	112.032	99.145
Total	832	93,993.302		112.998	100.000
**Model B**					
Year	1	102.038	102.038	− 0.162	− 0.154
Location	12	1,409.908	117.492	0.923	0.875
Error	181	18,947.900	104.685	104.684	99.278
Total	194	20,459.846		105.445	100.000

In Model A, all 59 populations collected from four *Brassica* host types in 2014 and 2015 were analyzed and variance was partitioned among years, among host within years and among populations within host. In Model B, populations from seven locations sampled in both 2014 and 2015 were analyzed and variance was partitioned among years and among locations within years.

**Figure 3:**
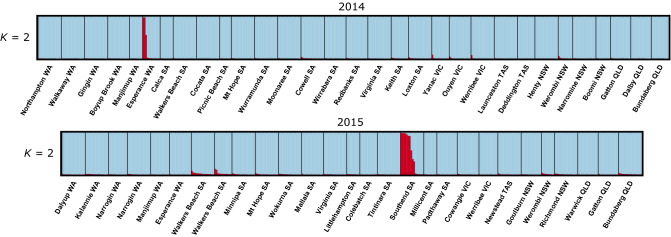
Proportional assignment to genotypic clusters, *K*, based on STRUCTURE analysis of *P. xylostella* individuals from Australia in 2014 and 2015. Individuals are represented by vertical bars and genotypic clusters are represented by different colours. Individuals collected each year were analysed separately and in both years the data most likely formed two genotypic clusters. Top panel: Analysis at *K* = 2 for 434 individuals collected from 31 locations in 2014. Bottom panel: Analysis at *K* = 2 for 399 individuals collected from 28 locations in 2015. Within years, bar plots show a high degree of genotypic admixture across individuals regardless of geographic location, as shown by sharing of blue-coloured bars, with a second genotypic cluster represented by red-coloured bars shared predominantly by several individuals at a single location.

Population structure was explored using two different individual-based clustering approaches. First, STRUCTURE analysis was performed using the widely-dispersed 1,032 SNPs and analysing 2014 and 2015 populations separately. We first determined the predicted optimal values for *K*, then examined bar plots for several *K* values to assess hierarchical population structure. In 2014, the data most likely formed two genotypic clusters, with the delta *K* method and mean likelihood value both producing an optimal at *K* = 2 (Supplemementary Fig. S4). At this *K* value, bar plots showed that most individuals shared nearly uniform ancestry across the major genotypic cluster regardless of geographic location (Fig. 3). A second genotypic cluster was largely associated with three individuals from Esperance, which showed 98.7%, 98.7% and 56.5% cluster assignment, while of the remaining 396 individuals, only 17 individuals were greater than 1% (1.0 to 9.3%) admixed across this cluster. At *K* = 3 and *K* = 4, no significant additional population structure was detected, with the additional genotypic clusters associated with two individuals from Boyup Brook and two individuals from Cocata (Supplementary Fig. S5).

In 2015, the delta *K* method produced an optimal at *K* = 2 and weaker secondary modes at *K* = 3 and *K* = 5 while the highest log-likelihood occurred at *K* = 5 (Supplementary Fig. S4). The modes at *K* = 3 and *K* = 5 indicate sub-structure in the data. At *K* = 2, most individuals shared nearly uniform ancestry across the major genotypic cluster regardless of geographic location (Fig. 3). The second genotypic cluster was predominantly associated with individuals from Southend, where 10 individuals showed 31.7 to 99.4% cluster assignment. At higher *K* values, further geographic structure was identified. At *K* = 3, two clusters were mainly associated with Southend (cluster A, 7 individuals with 26.1–98.6% assignment; cluster B: 10 individuals with 33.2–99.5% assignment) (Supplementary Fig. S5). At *K* = 4, the additional cluster was mainly associated with individuals collected from Walkers Beach in both autumn and spring 2015, showing a consistent pattern at both time points. At *K* = 5, the additional cluster was mostly represented by three individuals from Werombi. To further examine hierarchical structure, we reanalysed the 2015 data after removing Southend. This resulted in a weak delta *K* optimal at *K* = 3, but showed the same clustering pattern as the full 2015 dataset at *K* = 5 and is not presented.

**Figure 4:**
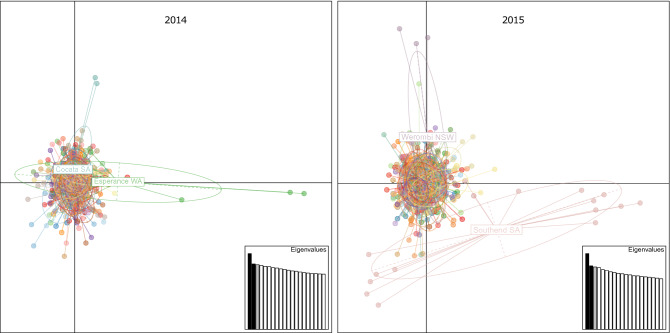
Principal components analysis of *P. xylostella* individuals collected from Australia in 2014 and 2015. Individuals are represented by small circles colour-coded by geographic population. Two populations with the most divergent individuals in each year are labelled.

Individual-based PCA analysis identified clustering patterns consistent with the STRUCTURE analysis. In both years, eigenvalues for the first principle component (PC) were not strongly different from eigenvalues for other PCs, indicating no clear axis of variance in the data, and individuals across different geographic populations clustered together to a high degree (Fig. 4). In both years, PCA identified the most divergent individuals consistent with those in the STRUCTURE analysis. In 2014, three individuals from Esperance and two individuals from Cocata clustered distinctly along separate PC axes. In 2015, two groups of individuals from Southend clustered distinctly along the two PCs axes, and three individuals from Werombi formed an identifiable cluster along the vertical PC axis.

## Discussion

In successful invading species, colonizing populations often exhibit reduced genetic diversity compared to their population of origin^53^. Previous molecular studies found a lack of genetic structure^19^ or inconsistent patterns of genetic structure^25,36^ among Australian populations of *P. xylostella*, and low genetic diversity implied a bottleneck during colonization^19,31^. To elucidate the movement patterns of *P. xylostella* in Australian canola cropping systems, we performed a comprehensive study of genetic structure among *P. xylostella* populations from crop and non-crop brassicaceous host plants throughout southern Australia. The study design included extensive field sampling to reflect the dispersal ecology of the species, molecular species identification, minimisation of sex bias, and a powerful SNP marker set derived from RAD-seq.

Genome-wide analysis revealed a distinct lack of genetic structure among Australian *P. xylostella* populations, irrespective of geographic location, host plant, or sampling year. This pattern was temporally stable at seven locations sampled in both 2014 and 2015. Our findings based on SNPs were highly consistent with those based on six microsatellites^19^. Our SNP-based estimates of global $F_{\text {ST}}$ within Australia were 0.0027 in 2014 and 0.0056 in 2015, compared to 0.0051 from microsatellites^19^. In both studies, > 99% of genetic variance occurred within populations with negligible variance explained by different locations or host plants. There was no evidence for isolation by distance, implying that any two populations, whether separated by distances of only several km or > 3,000 km, may be equally differentiated. Australian *P. xylostella* forms a single homogeneous population across bi-allelic neutral SNP markers.

Cluster analysis confirmed the overall lack of genetic divergence among populations. In both years, STRUCTURE analysis identified *K* = 2 as the most likely number of genotypic clusters. This is a common result among studies employing the delta *K* method^54^, because *K* = 1 cannot be obtained and because *K* = 2 often represents the top level of hierarchical population structure. At *K* = 2, a small number of divergent samples were identified at single geographic locations, including Esperance in 2014 and Southend in 2015. In 2015, at *K* values $\geqslant$ 3, two genotypic clusters occurred predominantly within the Southend population. Do these admixture patterns reflect genetic isolation? STRUCTURE sorts groups into Hardy–Weinberg linkage populations under the assumption of independent loci^55,56^. Among all populations, Southend had the lowest gene diversity, the fewest private sites and the highest $F_{\text {ST}}$ values in pairwise population comparisons. Notably, this population was collected from a small and isolated patch of sea rocket consisting only of several large plants. It is likely that cluster patterns reflect an artefact of sampling related individuals at Southend^57,58^. In hierarchical STRUCTURE analysis of 2014 and 2015 samples, additional genotype clusters at successively higher *K* values occurred in a small number of individuals from single locations as STRUCTURE simply grouped the next most related samples at each hierarchical level. These results highlight the need for caution when samples of related individuals are present, to avoid false inferences of population structure.

There is no evidence to suggest divergent samples represent interspecific hybrids of *P. xylostella* and the sympatric species, *P. australiana*. Although these species can hybridize in laboratory crosses, whether hybridization occurs in the wild is unknown^35^. Whole genome analysis of 29 *Plutella* individuals found no evidence for widespread introgression between these two species^59^. In our study, all Esperance and Southend individuals exhibited levels of heterozygosity across > 550,000 variant and invariant genome-wide loci that were similar to other *P. xylostella*. We would expect substantially higher heterozygosity if individuals were interspecific hybrids or if DNA samples were contaminated. PCA analysis of *P. xylostella* from Esperance 2014, Southend 2015 and five other locations, together with five *P. australiana* populations from Perry et al.^35^, showed clear species groupings and no evidence of introgressed individuals (Supplementary Fig. S6).

Genetic variation within a species is shaped by historical and contemporary evolutionary processes^7,60^. Because genetic homogeneity among Australian *P. xylostella* could reflect common ancestry^18^, present gene flow patterns are not clear from our data. Considering the vast size of the continent, it seems unlikely that *P. xylostella* forms a panmictic population in Australia in the sense that interbreeding is completely random. Saw et al.^31^ reported a small degree of sub-population structure among *P. xylostella* from 14 Australian locations based on geographic variation in frequencies of the dominant mtDNA haplotype. Insecticide resistance profiles of *P. xylostella* can vary spatially when the intensity of insecticide use differs across locations and host plant types, indicating that gene flow is often insufficient to overwhelm the effects of local selection on frequencies of resistance alleles^6,29^. Mo et al.^23^ found that *P. xylostella* moves over limited distances within actively growing *Brassica* vegetable crops where suitable host plants are continuously available. There is little propensity to emigrate during summer when crops are irrigated and the surrounding landscape is dry. Limited movement in these crops can lead to functional isolation of sub-populations, whereby resistance is selected by the insecticide spray regimes of individual farmers. Local selection, rather than spread of resistance alleles through gene flow, largely explained variation in levels of resistance to synthetic pyrethroids among Australian *P. xylostella* populations^6^.

Genetic differentiation indices are likely to over-estimate rates of gene flow, to the extent that genetic uniformity among *P. xylostella* reflects a genetic bottleneck and range expansion during colonization of Australia^61^. Although outgroups from other continents were unavailable for comparison, our recent data support the view that *P. xylostella* within Australia displays reduced genomic diversity compared to populations elsewhere. Australian *P. xylostella*, including 47 individuals from our study, were previously shown to exhibit 1.5-fold lower levels of heterozygosity across the nuclear genome relative to the endemic congeneric species, *P. australiana*^35^. Similarly, a study of microsatellite variation reported lower nucleotide diversity within *P. xylostella* populations from Australia than populations from Kenya and Indonesia^19^. *Plutella xylostella* exhibits lower mtDNA haplotype diversity within Australia^26,31,34,35^ than within parts of Asia, Africa, Europe and North America^10,26–28,31,34,62^. Analysis of mtDNA in 102 Australian *P. xylostella* individuals, including 44 individuals from our study, identified only five closely-related COI haplotypes (613 bp) and two dominant shared haplotypes^35^. Reduced diversity across the genome is strong evidence for a bottleneck^63^. By contrast, a recent global study of *P. xylostella* whole genomes reported unexpectedly high levels of SNP diversity within populations from the Oceania region, including Australia, New Zealand, Vanuatu and Samoa, compared to populations from putative historical source regions in the Americas, Europe, Africa and Asia^64^. Whether loci under selection may explain this pattern warrants further study. *Plutella xylostella* within Australia and New Zealand appears to have been founded by a small number of females derived from an ancestral lineage in southern Asia^26,31,34,35,64^.

It is likely that genetic homogeneity across the Australian *P. xylostella* distribution is maintained by some level of ongoing gene flow. RAD-seq markers revealed weak but significant genetic differentiation among field and laboratory-reared Australian *P. xylostella* populations ($F_{0}$ to $F_{6}$)^50^ but not wild populations ($F_{0}$, this study), implying that intermixing prevents divergence. Neutral bi-allelic SNPs failed to reveal the scale, frequency and timing of gene flow. Even very few migrants per generation can eliminate genetic differentiation among populations^65,66^, especially where genetic diversity is low. If a small founding *P. xylostella* population originally colonized Australia^31^, its present distribution throughout Australia demonstrates past gene flow at a continental level. This is consistent with the wide distribution of two shared haplotypes and with *P. xylostella* being a migratory species^4,13^. Within Australia, there is ample indirect evidence of *P. xylostella* dispersal, including the seasonal widespread colonization of winter-grown canola crops^18,67^ and detection of moth flights in light trapping and pheromone trapping studies^19,37,67–69^. In canola-growing areas remote from *Brassica* vegetable production, the annual canola cropping cycle of crop planting, senescence and harvest forces *P. xylostella* to disperse regularly between crops and wild brassicaceous host plants in the landscape. This tends to homogenise the insecticide resistance profiles of *P. xylostella* across Australian canola-growing regions, and among canola crops and *Brassica* weeds within each region^24^.

Gene flow creates potential for the spread of resistance alleles within and among Australian *Brassica* cropping systems. For certain newer insecticide chemistries, elevated resistance levels occur in *P. xylostella* within intensively sprayed *Brassica* vegetable crops relative to insects from canola crops or weeds^6^. Emigration of resistant moths from these cropping areas could contribute to the risk of *P. xylostella* insecticide resistance in canola cropping systems. Conversely, seasonal flights of *P. xylostella* moths into *Brassica* vegetable crops during spring^32,68^, perhaps originating from senescing canola crops or weeds, could dilute resistance levels if immigrants are more susceptible, and provide opportunities for rotation strategies to manage resistance. Insecticide-resistant *P. xylostella* genotypes can persist locally in Australian canola-growing areas where summer-active brassicas occur^67^. Evidence for large-scale gene flow among Australian *P. xylostella* suggests insecticide resistance alleles arising in one location could readily spread to other locations. These alleles may be selected to a high frequency in local areas if insecticides are used repeatedly^70^.

Neutral genome-wide SNPs were uninformative in identifying the dispersal patterns of *P. xylostella* in Australia, confirming previous conclusions^19^. Whether larger marker sets derived from massively parallel sequencing can provide insights into seasonal migration of *P. xylostella* in other global regions, where the species displays higher genetic diversity, remains to be evaluated.

## Methods

### Sample collection

Immature life stages (larvae or pupae; rarely, eggs) of *Plutella* species were collected between March 2014 and December 2015 throughout agricultural areas of southern Australia (Fig. 1). The dryland cropping areas of Australia experience climatic conditions most likely to support year-round persistence of *P. xylostella*^67,71^, and therefore represent the gene pool. Four brassicaceous *P. xylostella* host plant types were sampled: canola crops, *Brassica* vegetable and forage crops, and wild brassicaceous species (Fig. 1). The wild species were wild radish, *Raphanus raphanistrum*, turnip weed, *Rapistrum rugosum*, sea rocket, *Cakile maritima*, Ward’s weed, *Carrichtera annua*, and mixed stands of sand rocket, *Diplotaxis tenuifolia* and wall rocket, *D. muralis* (Table 1). In both years, most sampling was temporally restricted to periods in autumn (March to April) and spring (September to October) to minimise potential for migration to affect genetic structure^7^. Spring sampling corresponds to population peaks of *P. xylostella* in crops while autumn sampling corresponds to population troughs at the end of the summer/autumn non-cropping period, when host plants and the insect are often locally rare. Several locations were sampled in both years to allow temporal comparisons. At each location, $\geqslant$ 25 individuals were collected from multiple plants across a representative area to minimise sampling of related individuals, either using a sweep net in canola crops, *Brassica* forage crops and wild host plants, by hand in *Brassica* vegetable crops, or by beating plants over a collection tray for sea rocket. To eliminate parasitised individuals, each population was reared separately in a ventilated plastic container on leaves of the original host plant for 1–2 days and thereafter on cabbage leaves. Non-parasitised pupae or late-instar larvae were fresh frozen at − 80 °C.

### DNA isolation and species identification

For each population, 16 individuals were sequenced where possible after removing parasitised individuals. To avoid biases due to sex-linked markers^72^, we visually determined the sex of individual pupae (but not larvae) by examining external genital morphology^73^ under a dissecting microscope, then male and female individuals were selected to achieve a balanced sex ratio within each population where possible. Genomic DNA was isolated by homogenising whole individuals using a TissueLyser II (Qiagen) followed by two phenol and one chloroform extractions according to Zraket et al.^74^. DNA was treated with RNase A, then precipitated and resuspended in TE buffer. To distinguish *P. xylostella* from *P. australiana*, species identification was performed using a PCR-RFLP assay^35^ and *P. xylostella* individuals were retained for analysis.

### RAD-seq library preparation and sequencing

Libraries were prepared for restriction-site-associated DNA sequencing (RAD-seq) according to a protocol modified from Baird et al.^41^ as described in Perry et al.^35^. Genomic DNA was quantified using a Qubit 2.0 fluorometer (Invitrogen) and 200ng digested with 10 units of high fidelity *Sbf1* in Cutsmart buffer (NEB) for 1 h at 37 °C, then heat inactivated at 80 °C for 20 min. One microlitre of P1 adapter (100nM) with a 6-base molecular identifier (MID) (top strand 5$^{\prime }$-TCGTCGGCAGCGTCAGATGTGTATAAGAGACAGxxxxxxTGCA-3$^{\prime }$, bottom strand 5$^{\prime }$-[P]xxxxxxCTGTCTCTTATACACATCTGACGCTGCCGACGA-3$^{\prime }$, x represents sites for MIDs) were then added using 0.5$\upmu$L T4 DNA ligase (Promega), 1nM ATP and Cutsmart buffer. Sixteen individuals with unique P1 adapters were pooled per library. To minimise sequencing biases or batch effects, individuals from each population were randomised across 2–4 (usually 4) libraries and each library was sequenced across 2–4 sequencing lanes. Library pools were sheared using a Bioruptor sonicator (Diagenode), ends repaired using a Quick Blunting Kit (NEB), adenine overhangs added then P2 adapters (top strand 5$^{\prime }$-[P]CTGTCTCTTATACACATCTCCAGAATAG-3$^\prime$, bottom strand 5$^\prime$-GTCTCGTGGGCTCGGAGATGTGTATAAGAGACAGT-3$^{\prime }$) ligated, then (majority of libraries) size-selected (300–700 bp) on agarose gel to remove primer dimer. DNA purification between steps was performed using a MinElute PCR purification kit (Qiagen). Library amplification was performed using KAPA HiFi Hotstart Readymix (Kapa Biosystems) and Nextera i7 and i5 indexed primers with PCR conditions as described in Perry et al.^35^: 95 °C for 3 min, two cycles of 98 °C for 20 s, 54 °C for 15 s, 72 °C for 1 min, then 15 cycles of 98 °C for 20 s, 65 °C for 15 s, 72 °C for 1 min followed by a final extension of 72 °C for 5 min. Libraries were size-selected (300–700 bp) on agarose gel and purified using a minElute Gel Extraction Kit (Qiagen). Illumina paired-end sequencing was performed across seven lanes using HiSeq2500 (100 bp) or NextSeq500 (75 bp) at the Australian Genome Research Facility (AGRF). Additionally, 16 individuals from a separate sequencing run as described in Perry et al.^50^ were included in downstream analysis.

### Read filtering and variant calling

Sequence read quality was examined using FastQC^75^. As Nextseq reads had low quality base calls within restriction sites (a common problem when using fixed-length MIDs on this platform, which cause low sequence diversity and cluster signal in this region), we opted to remove restriction sites from all reads for downstream analysis. Sequence reads were de-multiplexed using RADtools version 1.2.4^42^ allowing one base MID mismatch, then TRIMMOMATIC v0.32^76^ was used to remove restriction sites, adapter sequences, a thymine base from reverse reads introduced by the P2 adapter, and quality filter using the ILLUMINACLIP tool with parameters: TRAILING:10 SLIDINGWINDOW:4:15 MINLEN:40. Paired reads were aligned to the *P. xylostella* reference genome (accession number: GCF_000330985.1) using STAMPY version 1.0.21^77^ with –baq and –gatkcigarworkaround options and expected substitution rate set to 0.03 to reflect our expectations of sequence divergence from the reference strain. Duplicate reads were removed and individual sample BAM files merged using PICARD version 1.71^78^. Genotypes were jointly called for all individuals using the Genome Analysis Tool Kit version 3.3-0^79,80^ HaplotypeCaller tool. We determined that base quality score recalibration using bootstrapped SNP databases was inappropriate for this dataset as it globally reduced quality scores. The variant call set was hard-filtered using VCFtools version 0.1.12a^81^. After iteratively testing multiple filtering parameter sets, we removed indels and retained confidently called bi-allelic SNPs (GQ $\geqslant$ 30) genotyped in at least 80% of individuals with a minimum genotype depth of 5, minQ $\geqslant$ 400, average site depth of 12–100, minimum minor allele frequency of 0.01 and in Hardy–Weinberg equilibrium at an alpha level of 0.05. To avoid closely-linked sites, we retained only SNPs separated by a minimum of 2,000 bp using the VCFtools—thin function. In order to estimate population-level genetic diversity, from the output of GATK HaplotypeCaller we generated a set of all confidently-called (GQ $\geqslant$ 30) variant and invariant sites and hard filtered to remove sites within repetitive regions and retain sites genotyped in at least 80% of individuals with an average site depth of 12–100. The filtered VCFs were converted to other file formats for downstream analysis using PGDSpider version 2.1.1.2^82^ and custom R scripts^83^.

### Genetic diversity

The R package hierfstat^84^ was used to calculate within-population gene diversity ($H_{\text {S}})$, observed heterozygosity ($H_{\text {O}}$) and the inbreeding coefficient ($F_{\text{IS}}$) according to Nei^85^. Population means for site depth and number of SNPs, indels and private sites were calculated using the –depth function and vcfstats module in VCFtools version 0.1.12a^81^.

### Population differentiation

To examine population differentiation, a global estimate of $F_{\text {ST}}$^86^ with bootstrapped 99% confidence intervals ($10^4$ bootstrap iterations) was calculated in R package diveRsity^87^. Pairwise $F_{\text {ST}}$ values for all population pairs were calculated and significance of differentiation determined using exact *G* tests ($10^4$ MCMC burnins, $10^3$ batches, $10^4$ iterations per batch) in GENEPOP v4.6^88^ after correction for multiple comparisons using the Bonferroni–Holm correction method^89,90^. Isolation by distance among populations^52^ was investigated separately for 2014 and 2015 datasets. We used R^91^ to construct heat maps and visually inspected the congruence between pairwise matrices of untransformed geographic distances in kilometres and genetic distances, $F_{\text {ST}}$, for corresponding population pairs. Significance of the regressions of pairwise linearized genetic distances^92^ onto log-transformed geographic distances was determined using a Mantel test with $10^4$ permutations in R package ade4 version 1.7-6^93^. Geographic distances were calculated using R package geosphere version 1.5-7^94^. Analysis of molecular variance (AMOVA) was performed using the pegas implementation in R package poppr version 2.7.1^95^. The data were analysed under two hierarchical model structures. In model A, all individuals were analysed together and populations were grouped into sampling years and *Brassica* host types. In Model B, a temporal analysis was performed for locations sampled in both 2014 and 2015, to investigate whether variance was greater among years within locations or vice versa.

### Population structure

Two individual-based clustering approaches were used to investigate population structure. First, Bayesian clustering was implemented in the program STRUCTURE version 2.3.4^55^. Variant data were converted from VCF to STRUCTURE file format using PDGSpider version 2.1.1.2^82^. For all runs, we used a burnin length of $5\times 10^5$ followed by a run length of 10$^6$ MCMC iterations and performed fifteen independent runs for each *K* value, where *K* is the number of genotypic clusters, using a different random seed for each run, assuming the *locprior* model with correlated allele frequencies and $\lambda$ set to 1. As preliminary runs showed that most structure was identified at low *K* values, we analysed *K*-values from 1 to 10 in both years. The optimal value of *K* was estimated using the delta *K* method^96^ implemented in STRUCTURE HARVESTER^97^ and inspection of the likelihood distribution for each model. *Q*-matrices were aligned using CLUMPP version 1.1.2^98^ and visualised using DISTRUCT version 1.1^99^. To further explore clustering, we performed individual-based principal components analysis (PCA) separately for 2014 and 2015 datasets using R package adegenet version 2.0.1^100,101^, using scaled and centred allele frequencies and imputing missing data by taking the mean of population allele frequencies.

### Power analysis

The statistical power of the SNP marker set to detect population structure was assessed using POWSIM version 4.1^102^. This program allows the user to test the likelihood of loci of detecting genetic differentiation for pre-defined values of $F_{\text {ST}}$. For the dataset, 1,000 simulations were performed over a range of $F_{\text {ST}}$ values from 0.001 to 0.01 assuming an effective population size of 5,000. The number of subpopulations, sample sizes and allele frequencies from our data were used and the generations of drift varied to achieve the target $F_{\text {ST}}$. As POWSIM currently handles a maximum of 30 populations, for the 2014 dataset the number of subpopulations was set to this value. The null hypothesis of genetic homogeneity was tested using Fisher’s exact test and a Chi-square test.

### Accession codes

RAD sequences are available from the Sequence Read Archive under accession PRJNA471964.

## Supplementary information

Supplementary Information.

## References

[1] Zalucki MP, Furlong MJ (2005). Forecasting *Helicoverpa* populations in Australia: a comparison of regression based models and a bioclimatic based modelling approach. Insect Sci..

[2] Mazzi D, Dorn S (2012). Movement of insect pests in agricultural landscapes. Ann. Appl. Biol..

[3] Eigenbrode SD (2016). Host-adapted aphid populations differ in their migratory patterns and capacity to colonize crops. J. Appl. Ecol..

[4] Furlong MJ, Wright DJ, Dosdall LM (2013). Diamondback moth ecology and management: problems, progress, and prospects. Annu. Rev. Entomol..

[5] Downes S (2017). A perspective on management of *Helicoverpa armigera*: transgenic Bt cotton, IPM, and landscapes. Pest Manag. Sci..

[6] Endersby NM, Ridland PM, Hoffmann AA (2008). The effects of local selection versus dispersal on insecticide resistance patterns: longitudinal evidence from diamondback moth (*Plutella xylostella* (Lepidoptera: Plutellidae)) in Australia evolving resistance to pyrethroids. Bull. Entomol. Res..

[7] Broquet T, Petit EJ (2009). Molecular estimation of dispersal for ecology and population genetics. Annu. Rev. Ecol. Evol. Syst..

[8] Pelissie B, Crossley MS, Cohen ZP, Schoville SD (2018). Rapid evolution in insect pests: the importance of space and time in population genomics studies. Curr. Opin. Insect Sci..

[9] Parry HR (2019). A native with a taste for the exotic: weeds and pasture provide year-round habitat for *Nysius vinitor* (Hemiptera: Orsillidae) across Australia, with implications for area-wide management. Aust. Entomol..

[10] Wei SJ (2013). Genetic structure and demographic history reveal migration of the diamondback moth *Plutella xylostella* (Lepidoptera: Plutellidae) from the southern to northern regions of China. PLoS ONE.

[11] Hereward JP, Walter GH, DeBarro PJ, Lowe AJ, Riginos C (2013). Gene flow in the green mirid, *Creontiades dilutus* (Hemiptera: Miridae), across arid and agricultural environments with different host plant species. Ecol. Evol..

[12] Zalucki MP (2012). Estimating the economic cost of one of the worlds major insect pests, *Plutella xylostella* (Lepidoptera: Plutellidae): just how long is a piece of string?. J. Econ. Entomol..

[13] Li Z, Feng X, Liu S-S, You M, Furlong MJ (2016). Biology, ecology, and management of the diamondback moth in China. Annu. Rev. Entomol..

[14] Talekar NS, Shelton A (1993). Biology, ecology, and management of the diamondback moth. Annu. Rev. Entomol..

[15] Mosiane SM, Kfir R, Villet MH (2003). Seasonal phenology of the diamondback moth, *Plutella xylostella* (L.), (Lepidoptera: Plutellidae), and its parasitoids on canola, *Brassica napus* (L.), in Gauteng province, South Africa. Afr. Entomol..

[16] Dosdall, L. M., Mason, P. G., Olfert, O., Kaminski, L. & Keddie, B. A. The origins of infestations of diamondback moth, *Plutella xylostella* (L.), in canola in western Canada. In *The Management of Diamondback Moth and Other Crucifer Pests: Proceedings of the Fourth International Workshop* (eds Endersby, N. M. & Ridland, P. M.) 95–100 (The Regional Institute Ltd, Gosford, New South Wales, Australia, 2004).

[17] Dosdall LM, Soroka JJ, Olfert O (2011). The diamondback moth in canola and mustard: current pest status and future prospects. Prairie Soils and Crops J..

[18] Furlong MJ (2008). Ecology of diamondback moth in Australian canola: landscape perspectives and the implications for management. Aust. J. Exp. Agric..

[19] Endersby NM, McKechnie SW, Ridland PM, Weeks AR (2006). Microsatellites reveal a lack of structure in Australian populations of the diamondback moth, *Plutella xylostella* (L.). Mol. Ecol..

[20] ABARES. Australian commodity production statistics. *Australian Bureau of Agricultural and Resource Economics and Sciences***cat. no. 7113.0** (2017).

[21] Gu H, Fitt GP, Baker GH (2007). Invertebrate pests of canola and their management in Australia: a review. Aust. J. Entomol..

[22] Baker, G. J. Crucifer vegetable insecticide resistance management strategies and issues in Australia. In *The Sixth International Workshop on Management of the Diamondback Moth and Other Crucifer Insect Pests* (eds Srinivasan, R., Shelton, A. M. & Collins, H. L.) 21–25 (AVRDC – The World Vegetable Centre, Tainan, Taiwan, 2011).

[23] Mo JH, Baker G, Keller M, Roush R (2003). Local dispersal of the diamondback moth (*Plutella xylostella* (L.)) (Lepidoptera: Plutellidae). Environ. Entomol..

[24] Baker, G. J. Improving management of diamondback moth in Australian canola: final report for GRDC (DAS00134). Technical Report, South Australian Research and Development Institute (2015).

[25] Pichon A (2006). Genetic differentiation among various populations of the diamondback moth, *Plutella xylostella* (Lepidoptera: Yponomeutidae). B. Entomol. Res..

[26] Juric I, Salzburger W, Balmer O (2017). Spread and global population structure of the diamondback moth *Plutella xylostella* (Lepidoptera: Plutellidae) and its larval parasitoids *Diadegma semiclausum* and *Diadegma fenestrale* (Hymenoptera: Ichneumonidae) based on mtDNA. B. Entomol. Res..

[27] Kim I (2003). Mitochondrial COI gene sequence-based population genetic structure of the diamondback moth, *Plutella xylostella*, Korea. Korean J. Genet..

[28] Yang J (2015). Insight into the migration routes of *Plutella xylostella* in China using mtCOI and ISSR markers. PLoS ONE.

[29] Caprio MA, Tabashnik BE (1992). Allozymes used to estimate gene flow among populations of diamondback moth (Lepidoptera, Plutellidae) in Hawaii. Environ. Entomol..

[30] Chang WXZ (1997). Mitochondrial DNA sequence variation among geographic strains of diamondback moth (Lepidoptera: Plutellidae). Ann. Entomol. Soc. Am..

[31] Saw J, Endersby NM, McKechnie SW (2006). Low mtDNA diversity among widespread Australian diamondback moth *Plutella xylostella* (L.) suggests isolation and a founder effect. Insect Sci..

[32] Endersby, N. M. Population structure and gene flow in diamondback moth in Australia and around the world: current state of knowledge and directions for the future. In *The Management of Diamondback Moth and Other Crucifer Pests: Proceedings of the Fifth International Workshop* (eds Shelton A. M. *et al.*) 132–147 (China Agricultural Science and Technology Press, Beijing, 2008).

[33] Fu X, Xing Z, Liu Z, Ali A, Wu K (2014). Migration of diamondback moth, *Plutella xylostella*, across the Bohai sea in northern China. Crop Prot..

[34] Delgado AM, Cook JM (2009). Effects of a sex-ratio distorting endosymbiont on mtDNA variation in a global insect pest. BMC Evol. Biol..

[35] Perry KD (2018). Cryptic Plutella species show deep divergence despite the capacity to hybridize. BMC Evol. Biol..

[36] Roux O (2007). ISSR-PCR: tool for discrimination and genetic structure analysis of *Plutella xylostella* populations native to different geographical areas. Mol. Phylogenet. Evol..

[37] Landry JF, Hebert PDN (2013). *Plutella australiana* (Lepidoptera, Plutellidae), an overlooked diamondback moth revealed by DNA barcodes. Zookeys.

[38] Goodwin S, McPherson JD, McCombie WR (2016). Coming of age: ten years of next-generation sequencing technologies. Nat. Rev. Genet..

[39] Davey JW (2011). Genome-wide genetic marker discovery and genotyping using next-generation sequencing. Nat. Rev. Genet..

[40] Narum SR, Buerkle CA, Davey JW, Miller MR, Hohenlohe PA (2013). Genotyping-by-sequencing in ecological and conservation genomics. Mol. Ecol..

[41] Baird NA (2008). Rapid SNP discovery and genetic mapping using sequenced RAD markers. PLoS ONE.

[42] Baxter SW (2011). Linkage mapping and comparative genomics using next-generation RAD sequencing of a non-model organism. PLoS ONE.

[43] Andrews KR, Good JM, Miller MR, Luikart G, Hohenlohe PA (2016). Harnessing the power of RADseq for ecological and evolutionary genomics. Nat. Rev. Genet..

[44] Davey JL, Blaxter MW (2010). RADSeq: next-generation population genetics. Brief. Funct. Genomics.

[45] Putman AI, Carbone I (2014). Challenges in analysis and interpretation of microsatellite data for population genetic studies. Ecol. Evol..

[46] Haasl RJ, Payseur BA (2011). Multi-locus inference of population structure: a comparison between single nucleotide polymorphisms and microsatellites. Heredity.

[47] Vendrami DLJ (2017). RAD sequencing resolves fine-scale population structure in a benthic invertebrate: implications for understanding phenotypic plasticity. R. Soc. Open Sci..

[48] Rasic G (2015). *Aedes aegypti* has spatially structured and seasonally stable populations in Yogyakarta, Indonesia. Parasit. Vectors.

[49] Rasic G (2015). Contrasting genetic structure between mitochondrial and nuclear markers in the dengue fever mosquito from Rio de Janeiro: implications for vector control. Evol. Appl..

[50] Perry, K. D., Pederson, S. M. & Baxter, S. W. Genome-wide SNP discovery in field and laboratory colonies of Australian *Plutella* species. In *Proceedings of the Seventh International Workshop on Management of the Diamondback Moth and Other Crucifer Insect Pests,* *Mysore J. Agric. Sci.*, **51**A, 18–31 (2017).

[51] Fountain ED, Pauli JN, Reid BN, Palsboll PJ, Peery MZ (2016). Finding the right coverage: the impact of coverage and sequence quality on single nucleotide polymorphism genotyping error rates. Mol. Ecol. Resour..

[52] Wright S (1943). Isolation by distance. Genetics.

[53] Grapputo A, Boman S, Lindstrom L, Lyytinen A, Mappes J (2005). The voyage of an invasive species across continents: genetic diversity of North American and European Colorado potato beetle populations. Mol. Ecol..

[54] Janes JK (2017). The $$K=2$$ conundrum. Mol. Ecol..

[55] Pritchard J, Stephens M, Donnelly P (2000). Inference of population structure using multilocus genotype data. Genetics.

[56] Kalinowski ST (2011). The computer program STRUCTURE does not reliably identify the main genetic clusters within species: simulations and implications for human population structure. Heredity.

[57] Rodriguez-Ramilo ST, Wang J (2012). The effect of close relatives on unsupervised Bayesian clustering algorithms in population genetic structure analysis. Mol. Ecol. Resour..

[58] Wang J (2018). Effects of sampling close relatives on some elementary population genetics analyses. Mol. Ecol. Resour..

[59] Ward CM, Baxter SW (2018). Assessing genomic admixture between cryptic *Plutella* moth species following secondary contact. Genome Biol. Evol..

[60] Epps CW, Keyghobadi N (2015). Landscape genetics in a changing world: disentangling historical and contemporary influences and inferring change. Mol. Ecol..

[61] Donnelly M, Licht M, Lehmann T (2001). Evidence for recent population expansion in the evolutionary history of the malaria vectors *Anopheles arabiensis* and *Anopheles gambiae*. Mol. Biol. Evol..

[62] Niu YQ, Nansen C, Li XW, Liu TX (2014). Geographical variation of *Plutella xylostella* (Lepidoptera: Plutellidae) populations revealed by mitochondrial COI gene in China. J. Appl. Entomol..

[63] Hurst GDD, Jiggins FM (2005). Problems with mitochondrial DNA as a marker in population, phylogeographic and phylogenetic studies: the effects of inherited symbionts. Proc. R. Soc. B Biol. Sci..

[64] You M (2020). Variation among 532 genomes unveils the origin and evolutionary history of a global insect herbivore. Nat. Commun..

[65] Slatkin M (1985). Gene flow in natural populations. Annu. Rev. Ecol. Syst..

[66] Mills LS, Allendorf FW (1996). The one-migrant-per-generation rule in conservation and management. Cons. Biol..

[67] Perry, K. D. *The colonisation of canola crops by the diamondback moth, Plutella xylostella L., in southern Australia*. Ph.D. thesis, The University of Adelaide (2019).

[68] Hatami, B. *Seasonal occurrence and abundance of diamondback moth, Plutella xylostella (L.), and its major parasitoids on brassicaceous plants in South Australia*. Ph.D. thesis, The University of Adelaide (1996).

[69] Ridland, P. M. & Endersby, N. M. Seasonal phenology of diamondback moth populations in southern Australia. In *The Management of Diamondback Moth and Other Crucifer Pests: Proceedings of the Fifth International Workshop* (eds Shelton A. M. *et al.*) 90–101 (China Agricultural Science and Technology Press, Beijing, 2008).

[70] Roush RT, McKenzie JA (1987). Ecological genetics of insecticide and acaricide resistance. Annu. Rev. Entomol..

[71] Zalucki, M. P. & Furlong, M. J. Predicting outbreaks of a migratory pest: an analysis of DBM distribution and abundance revisited. In *The Sixth International Workshop on Management of the Diamondback Moth and Other Crucifer Insect Pests* (eds Srinivasan, R., Shelton, A. M. & Collins, H. L.) 8–14 (AVRDC – The World Vegetable Centre, Tainan, Taiwan, 2011).

[72] Benestan L (2017). Sex matters in massive parallel sequencing: evidence for biases in genetic parameter estimation and investigation of sex determination systems. Mol. Ecol..

[73] Robertson PL (1939). Diamondback moth investigation in New Zealand. N. Z. J. Sci. Technol..

[74] Zraket, C., Barth, J., Heckel, D. & Abbott, A. Genetic linkage mapping with restriction fragment length polymorphisms in the tobacco budworm, *Heliothis virescens. *In *Molecular Insect Science* (eds Hagedorn, H. H., Hildebrand, J. G., Kidwell M. G. & Law, J. H.) 13–20 (Springer, Boston, MA, 1990). 10.1007/978-1-4899-3668-4_2

[75] Andrews, S. FASTQC: a quality control tool for high throughput sequence data. http://www.bioinformatics.babraham.ac.uk/projects/fastqc/ (2010). Accessed August 2016.

[76] Bolger AM, Lohse M, Usadel B (2014). Trimmomatic: a flexible trimmer for Illumina sequence data. Bioinformatics.

[77] Lunter G, Goodson M (2011). Stampy: a statistical algorithm for sensitive and fast mapping of Illumina sequence reads. Genome Res..

[78] Broad Institute. http://broadinstitute.github.io/picard/. Accessed 10 December 2017.

[79] McKenna A (2010). The Genome Analysis Toolkit: a MapReduce framework for analyzing next-generation DNA sequencing data. Genome Res..

[80] DePristo MA (2011). A framework for variation discovery and genotyping using next-generation DNA sequencing data. Nat. Genet..

[81] Danecek P (2011). The variant call format and VCFtools. Bioinformatics.

[82] Lischer HEL, Excoffier L (2012). PGDSpider: an automated data conversion tool for connecting population genetics and genomics programs. Bioinformatics.

[83] Perry, K. D. https://github.com/kymperry01/PlutellaCanola. Accessed November 2018.

[84] Goudet, J. & Jombart, T. *hierfstat: Estimation and tests of hierarchical F-statistics* (2015). R package version 0.04-22.

[85] Nei M (1987). Molecular Evolutionary Genetics.

[86] Weir B, Cockerham C (1984). Estimating *F*-statistics for the analysis of population structure. Evolution.

[87] Keenan K, McGinnity P, Cross TF, Crozier WW, Prodoehl PA (2013). diveRsity: an R package for the estimation and exploration of population genetics parameters and their associated errors. Methods Ecol. Evol..

[88] Rousset F (2008). GENEPOP ‘007: a complete re-implementation of the GENEPOP software for Windows and Linux. Mol. Ecol. Resour..

[89] Holm S (1979). A simple sequentially rejective multiple test procedure. Scand. J. Stat..

[90] Armstrong RA (2014). When to use the Bonferroni correction. Ophthal. Physiol. Opt..

[91] R Core Team. *R: A Language and Environment for Statistical Computing*. R Foundation for Statistical Computing, Vienna, Austria (2017).

[92] Slatkin M (1995). A measure of population subdivision based on microsatellite allele frequencies. Genetics.

[93] Dray S, Dufour AB (2007). The ade4 package: implementing the duality diagram for ecologists. J. Stat. Softw..

[94] Hijmans, R. J. *geosphere: Spherical Trigonometry*. R package version 1.5-7 (2017).

[95] Kamvar ZN, Tabima JF, Gruenwald NJ (2014). Poppr: an R package for genetic analysis of populations with clonal, partially clonal, and/or sexual reproduction. PeerJ.

[96] Evanno G, Regnaut S, Goudet J (2005). Detecting the number of clusters of individuals using the software STRUCTURE: a simulation study. Mol. Ecol..

[97] Earl DA, von Holdt BM (2012). STRUCTURE HARVESTER: a website and program for visualizing STRUCTURE output and implementing the Evanno method. Conserv. Genet. Resour..

[98] Jakobsson M, Rosenberg NA (2007). CLUMPP: a cluster matching and permutation program for dealing with label switching and multimodality in analysis of population structure. Bioinformatics.

[99] Rosenberg NA (2004). DISTRUCT: a program for the graphical display of population structure. Mol. Ecol. Notes.

[100] Jombart T (2008). adegenet: an R package for the multivariate analysis of genetic markers. Bioinformatics.

[101] Jombart T, Ahmed I (2011). adegenet 1.3-1: new tools for the analysis of genome-wide SNP data. Bioinformatics.

[102] Ryman N, Palm S (2006). POWSIM: a computer program for assessing statistical power when testing for genetic differentiation. Mol. Ecol. Notes.

