# Supplementary material for: Genome-wide analysis of diamondback moth, Plutella xylostella L., from Brassica crops and wild host plants reveals no genetic structure in Australia
Source: Sci Rep. 2020 Jul 21;10:12047. doi: 10.1038/s41598-020-68140-w (PMC7374630; doi:10.1038/s41598-020-68140-w)
Supplement: Supplementary file 1 — Supplementary Information. [file 41598_2020_68140_MOESM1_ESM.pdf]

Supplementary Tables and Figures

Genome-wide analysis of diamondback moth, *Plutella xylostella* L., from *Brassica* crops and wild host plants reveals no genetic structure in Australia

Kym D. Perry[1,2]*, Michael A. Keller[2] and Simon W. Baxter[3]*

[1]University of Adelaide, School of Agriculture Food and Wine, Adelaide, 5005, Australia
[2]South Australian Research and Development Institute, Entomology Unit, Adelaide, 5001, Australia
[3]University of Melbourne, School of BioSciences, Melbourne, 3010, Australia

*Corresponding authors: kym.perry@adelaide.edu.au; simon.baxter@unimelb.edu.au

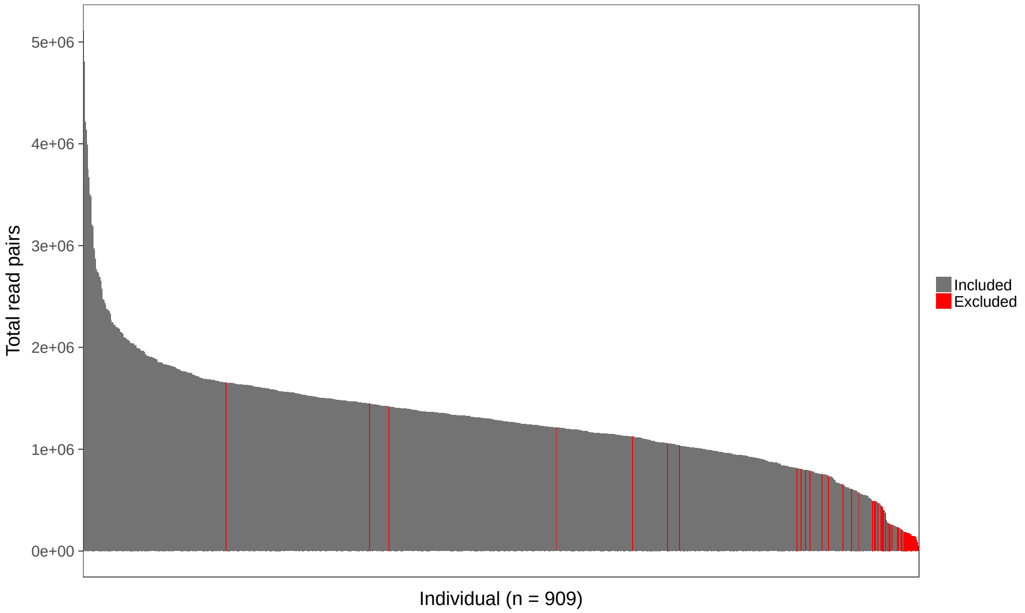

Figure S1: Bar plot showing the total number of sequence read pairs per *P. xylostella* individual (n = 909 individuals). Individuals coloured grey were included in the analysis while 50 individuals coloured in red were excluded due to greater than 60 % missing data across genotyped SNPs after initial hard filtering. For most excluded individuals, the missing data can be attributed to low sequencing depth.

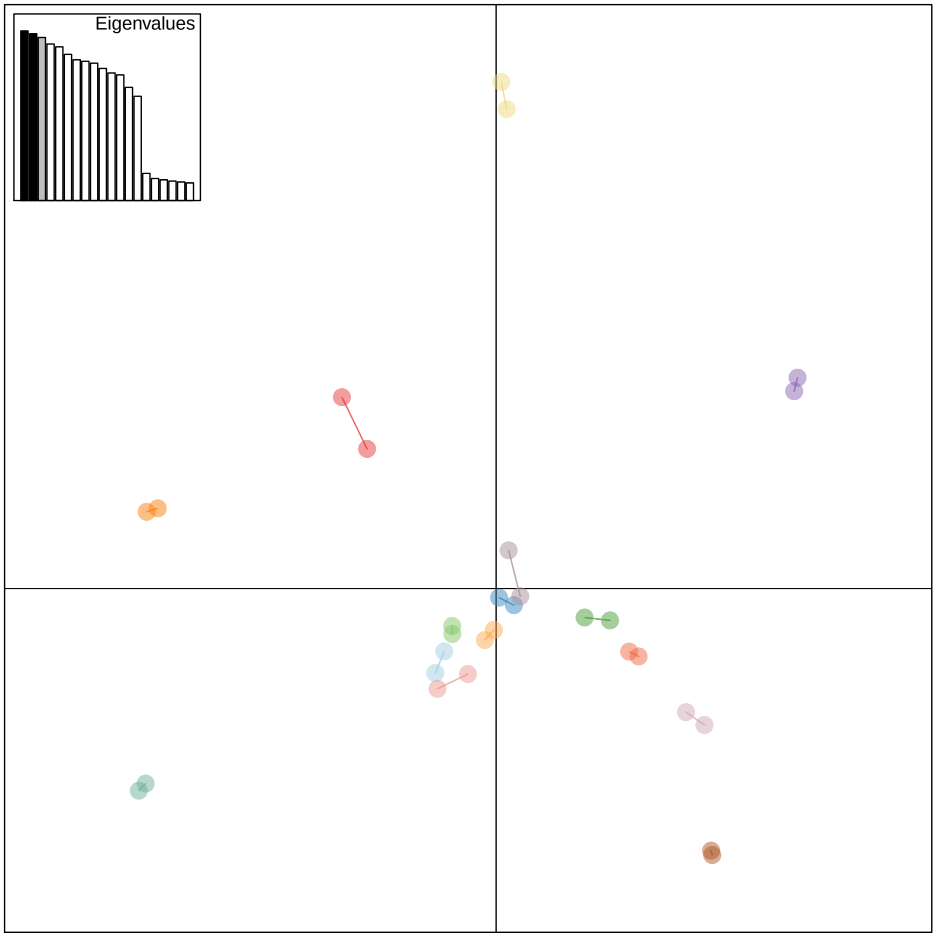

Figure S2: PCA for 15 individuals randomly selected from among the 906 individuals in our study, and sequenced and genotyped in duplicate to cross-check a representative sample of genotypes. Duplicate DNA samples from each individual were sequenced within different RAD-seq library pools, then genotyped using the same bioinformatics pipeline and parameters in our study. A total of 1473 SNPs genotyped in 80 % of individuals were used for the PCA with missing data imputed by taking the mean of allele frequencies. Paired genotypes based on DNA samples from the same individual are shown in circles of the same colour. Sample pairs group closely together, indicating that genotype calls were highly consistent.

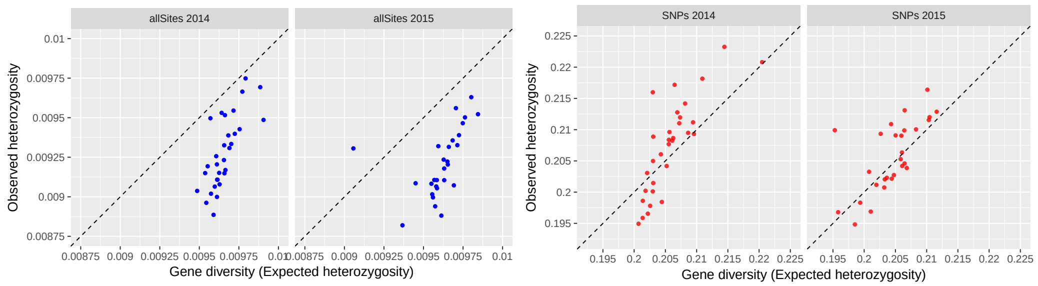

Figure S3: Population means for observed versus expected heterozygosity across 590 068 variant and invariant genome-wide sites (left panels, blue dots) and 1032 genome-wide SNPs (right panels, red dots) for *P. xylostella* populations collected from Australia in 2014 ($n = 31$ populations, 434 individuals) and 2015 ($n = 28$ populations, 399 individuals).

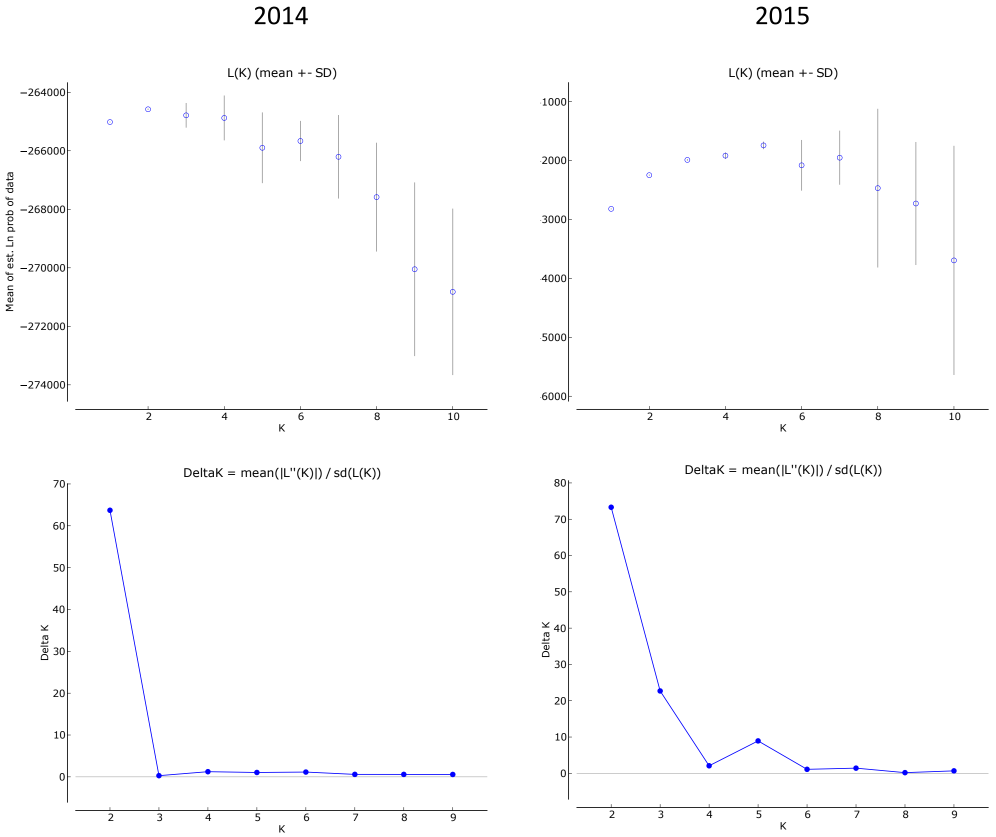

Figure S4: Structure Harvester[1] plots of mean log-likelihood ($L(K)$) and delta K from STRUCTURE analysis of Australian *P. xylostella*. Top panels: Mean log-likelihood ($L(K)$) value over 15 STRUCTURE runs for each K value for *P. xylostella* in 2014 (n = 31 populations, 434 individuals) and 2015 (n = 28 populations, 399 individuals). Bottom panels: Plot of delta K based on the same STRUCTURE analysis according to Evanno et al.[1]. In 2014, a clear mode occurs at $K = 2$. In 2015, a primary mode occurs at $K = 2$ and weaker secondary modes occur at $K = 3$ and $K = 5$.

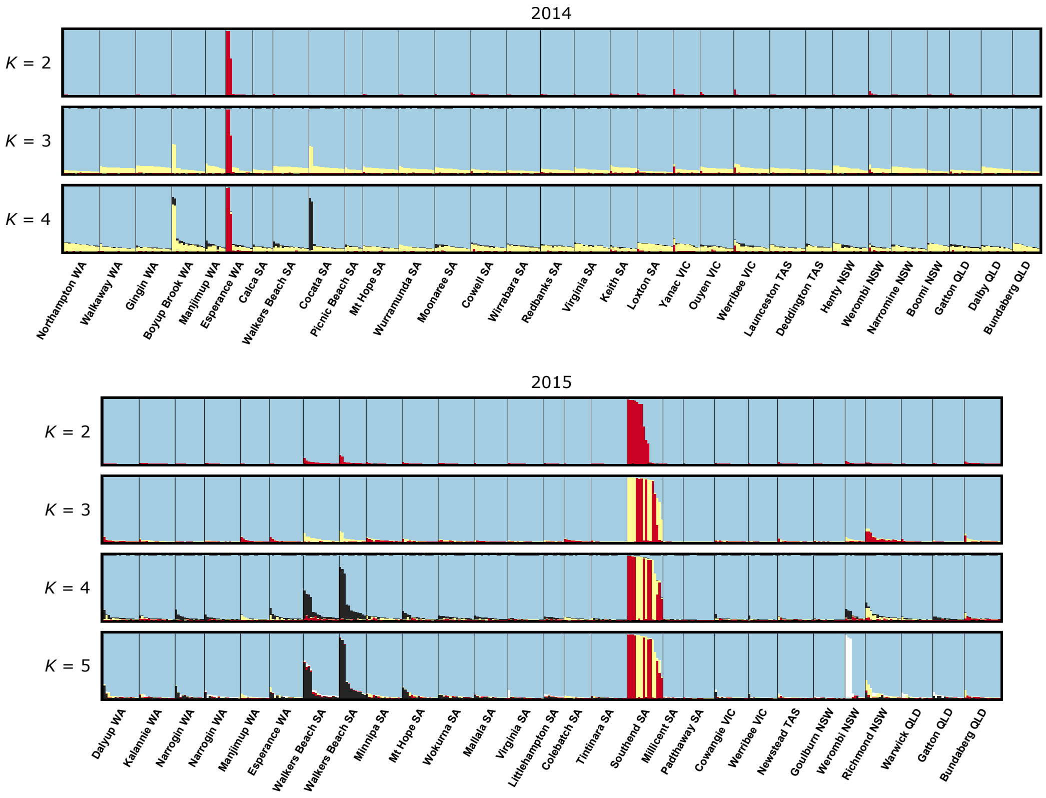

Figure S5: Proportional assignment to genotypic clusters, K, based on STRUCTURE analysis of *P. xylostella* individuals from Australia in 2014 and 2015 for $K = 2 - 5$. Individuals are represented by vertical bars and genotypic clusters are represented by different colours. Individuals collected from each year were analysed separately and in both years the data most likely formed two genotypic clusters. Top panel: Analysis at $K = 2 - 4$ for 434 individuals collected from 31 locations in 2014. Bottom panel: Analysis at $K = 2 - 5$ for 399 individuals collected from 28 locations in 2015.

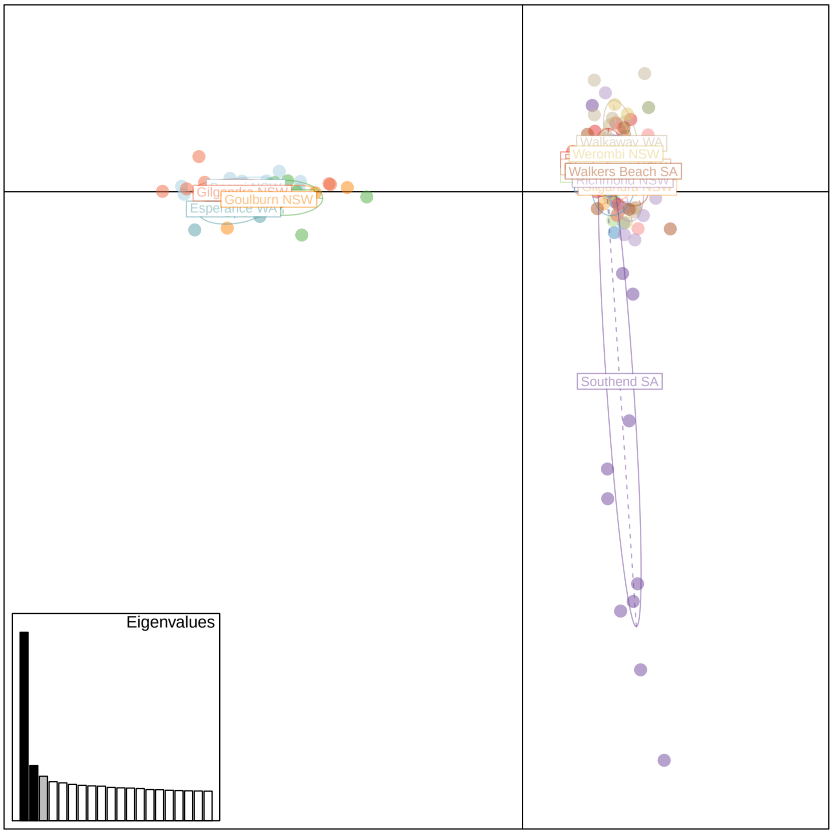

Figure S6: PCA of 200 *Plutella* individuals from 12 Australian locations to examine whether *P. xylostella* from Esperance 2014 and Southend 2015 include interspecific hybrid individuals. Included individuals were 47 *P. xylostella* and 52 *P. australiana* from Perry et al.[2] co-existing at five locations: Esperance 2014, Boomi 2014, Calca 2014, Gilgandra 2014, Goulburn 2015, and 101 additional *P. xylostella* from seven widely-separated locations: Southend 2015, Cocata 2014, Bundaberg 2015, Richmond 2015, Walkaway 2014, Walkers Beach 2015 (September), Werombi 2015. All individuals were joint-genotyped using GATK HaplotypeCaller and GenotypeGVCFs tools, hard-filtered using our standard filtering parameters, then PCA was performed based on 1164 bi-allelic SNPs following methods described in the manuscript. Individuals form clear species groups along eigenvector1 (horizontal axis) and this explains most variance, as shown by eigenvalues. All *P. australiana* cluster in the left two quadrants and all *P. xylostella* in the right two quadrants. There is no evidence of interspecific hybrid individuals. Southend individuals explain most variance along eigenvector2.

Table S1: Power analysis for 1032 SNP marker loci identified in Australian populations of *P. xylostella*. Presented are the probabilities of SNP loci detecting true population differentiation at predefined F_{ST} values according to Fisher's Exact and Chi-Squared tests. Analyses were conducted in POWSIM assuming an effective population size (N_e) of 5000. Simulations for $F_{\text{ST}} = 0.0027$ were conducted for 2014 data only and $F_{\text{ST}} = 0.0056$ for 2015 data only, corresponding to global F_{ST} estimates for these years.

	2014		2015	
F_{ST}	Fisher's Exact	Chi-Square	Fisher's Exact	Chi-Square
0.0010	0.9280	0.9580	0.9200	0.9490
0.0027	1.0000	1.0000	–	–
0.0050	1.0000	1.0000	1.0000	1.0000
0.0056	–	–	1.0000	1.0000
0.0100	1.0000	1.0000	1.0000	1.0000

References

[1] Evanno G, Regnaut S, Goudet J. Detecting the number of clusters of individuals using the software STRUCTURE: A simulation study. Mol Ecol. 2005 Jul;14(8):2611–2620.

[2] Perry KD, Baker GJ, Powis KJ, Kent JK, Ward CM, Baxter SW. Cryptic *Plutella* species show deep divergence despite the capacity to hybridize. BMC Evol Biol. 2018 May;18.
